# Extremely high proportions of male flowers and geographic variation in floral ratios within male figs of *Ficus tikoua* despite pollinators displaying active pollen collection

**DOI:** 10.1002/ece3.1926

**Published:** 2016-01-09

**Authors:** Jun‐Yin Deng, Rong‐Hua Fu, Stephen G. Compton, Dai‐Mei Hu, Lu‐Shui Zhang, Fan Yang, Yan Chen, Finn Kjellberg

**Affiliations:** ^1^ Ecological Security and Protection Key laboratory of Sichuan ProvinceCollege of Life Science and BiotechnologyMianyang Normal UniversityMianyangSichuan621000China; ^2^ School of BiologyUniversity of LeedsLeedsLS2 9JTUK; ^3^ Department of Zoology and EntomologyRhodes UniversityGrahamstown6140South Africa; ^4^ CEFE UMR 5175CNRSUniversité de MontpellierUniversité Paul‐Valéry MontpellierEPHEMontpellierFrance

**Keywords:** Active pollination, anther‐to‐ovule ratios, *Ceratosolen*, fig wasp, inflorescence design, P/O ratios, pollen limitation

## Abstract

Most plants are pollinated passively, but active pollination has evolved among insects that depend on ovule fertilization for larval development. Anther‐to‐ovule ratios (A/O ratios, a coarse indicator of pollen‐to‐ovule ratios) are strong indicators of pollination mode in fig trees and are consistent within most species. However, unusually high values and high variation of A/O ratios (0.096–10.0) were detected among male plants from 41 natural populations of *Ficus tikoua* in China. Higher proportions of male (staminate) flowers were associated with a change in their distribution within the figs, from circum‐ostiolar to scattered. Plants bearing figs with ostiolar or scattered male flowers were geographically separated, with scattered male flowers found mainly on the Yungui Plateau in the southwest of our sample area. The A/O ratios of most *F. tikoua* figs were indicative of passive pollination, but its *Ceratosolen* fig wasp pollinator actively loads pollen into its pollen pockets. Additional pollen was also carried on their body surface and pollinators emerging from scattered‐flower figs had more surface pollen. Large amounts of pollen grains on the insects' body surface are usually indicative of a passive pollinator. This is the first recorded case of an actively pollinated *Ficus* species producing large amounts of pollen. Overall high A/O ratios, particularly in some populations, in combination with actively pollinating pollinators, may reflect a response by the plant to insufficient quantities of pollen transported in the wasps’ pollen pockets, together with geographic variation in this pollen limitation. This suggests an unstable scenario that could lead to eventual loss of wasp active pollination behavior.

## Introduction

Around 80% of all flowering plant species require the services of insects for their sexual reproduction (Ghazoul 2005; Biesmeijer et al. 2006; Potts et al. 2010), making pollination of flowers by insects central to the maintenance of terrestrial biodiversity (Kearns et al. 1998; Biesmeijer et al. 2006). Seed production is often pollen‐limited, meaning that although plants have the resources to support production of more seeds, their ovules remain unpollinated (Wilcock and Neiland 2002; Ashman et al. 2004; Knight et al. 2005). Plants may therefore compete for pollen (Mitchell et al. 2009). In the longer term, this can generate selection pressures that result in the evolution and modification of traits related to both a plant's female and male reproductive functions (Larson and Barrett 2000; Fenster et al. 2004; Knight et al. 2005). This may affect how resources are allocated to male and female function by affecting inflorescence design, but not in a way that will maximize the efficiency of reproduction: the ESS trade‐off between male and female investment is a compromise in investment when marginal male and female function benefits become equal (Fishbein and Venable 1996). Potential responses to pollen limitation include increased investment in pollinator attraction and rewards, improved efficiency of pollen transfer by flower morphological optimization, the production of smaller, but more numerous, pollen grains, and an increased reliance on self‐pollination (Ashman et al. 2004; Gotzenberger et al. 2007; Harder and Aizen 2010).

In a small number of plant lineages, passive (topocentric) pollination has been replaced by active pollination (ethodynamic sensu Galil 1973), where pollinating insects both actively collect pollen and deposit pollen on receptive stigmas. This mode of pollination would seem to ensure particularly efficient pollen transport and plants present particular traits of pollen and stigma presentation associated with active pollination. Active pollination has evolved at least four times, involving *Senita* (Cactaceae) and its pollinating crambid moths (Fleming and Holland 1998), *Glochidion/Phyllanthus* (Phyllanthaceae) and gracillariid moths (Kato et al. 2003), *Yucca* (Asparagaceae) and prodoxid moths (Pellmyr et al. 1996), *Ficus* (Moraceae) and subfamilies of Agaonidae fig wasps other than Sycophaginae (Heraty et al. 2013). Although active pollination may appear particularly effective, it is only observed in cases of nursery pollination (Sakai 2002) and there are costs associated with the nutrition of pollinator offspring. In most nursery pollination systems, pollinator offspring do not transport pollen from their natal individual plant and as such are a cost to their host plant: they must be considered as parasites of the female function of the plant. However, in the *Ficus*–fig wasp pollination mutualism, female wasps transport pollen from their natal figs, and therefore benefit the male function of their host plant by dispersing its pollen (Anstett et al. 1997). This particular situation has allowed the evolution of reduced pollen production in actively pollinated fig species, a feature that probably allows the plants to produce more pollen vectors instead (Kjellberg et al. 2001). The balance between pollinator and anther production may nevertheless be suboptimal, resulting in what appears to be limiting pollen loads per wasp (Kjellberg et al. 2014).

Fig trees and their associated fig wasps represent one of the most specialized examples of obligate pollination mutualism. Fig trees are ecologically important components of tropical or subtropical communities, with about 800 species that display a diversity in life‐form (trees, shrubs, stranglers, and vines), breeding system (monoecy and dioecy), and pollination mode (active and passive pollination) (Herre et al. 2008; Chen et al. 2010). The interaction between fig trees and fig wasps centers on the figs, which are unique enclosed inflorescences containing many tiny flowers. Female fig wasps enter figs through a narrow ostiole in order to lay their eggs inside the female flowers, where the larvae will feed.

*Ficus* species are each pollinated by one or a few generally host‐specific species of fig wasps. Monoecious fig trees have consistently mutualistic relationships with their pollinators, where fig wasps that enter the figs both pollinate the flowers that line the inside and lay their eggs in some of them. More centrally located ovules, presenting shorter styles, tend to support the development of pollinator larvae, and more peripheral ovules, presenting longer styles, are more likely to develop into seeds (Nefdt and Compton 1996; Jousselin et al. 2001). Dioecious *Ficus* species have female individuals with figs that only produce seeds, and male individuals that support development of fig wasps in their female flowers and produce pollen for them to transport, but they produce no seeds.

Pollinator fig wasp larvae feed on developing endosperm. The adult females of some species are pollinating actively and mainly initiate endosperm development by ensuring the double fertilization of the fig ovule by depositing pollen on the stigmas, while other fig wasp species are passive pollinators that trigger parthenogenetic development of the endosperm, so that no fertilization of the ovule is required (Borges and Kjellberg 2014). Globally, about one‐third of fig tree species are pollinated passively by fig wasps, but pollination mode frequencies vary between lineages and hence between continents (Kjellberg et al. 2001). Females of passively pollinating fig wasps become dusted with pollen released from dehiscent anthers when they emerge from the galls within their natal figs. Pollination is achieved when the pollen is subsequently brushed off by the stigmas inside a second, receptive fig. Fig wasps that actively pollinate display more complex behavior. On emergence from their galls, they seek out anthers from which they transfer pollen into thoracic pollen pockets using their forelegs. Lines of hairs (‘coxal combs’) aid pollen collection. Once inside a receptive fig, the fig wasps remove pollen from the pockets and transfer it onto the stigmas, generally at the same time as they are laying their eggs.

The advantages of active pollination appear to be clear for the plants, which can produce less pollen and instead produce more pollen vectors, because pollen loading on the wasps and subsequent deposition is more efficient (Kjellberg et al. 2001). Similarly, actively pollinating insects are likely to benefit from active pollination behavior because it increases the likelihood of additional resources being available to their larvae (ovules with developing endosperm as opposed to unpollinated ovules in which they have to induce the parthenogenetic development of the endosperm) (Borges and Kjellberg 2014). For actively pollinating fig wasps, reduced numbers of offspring (‘sanctions’) have been recorded for experimental pollen‐free fig wasps introduced into figs where no other females had deposited pollen (Nefdt 1989; Jandér and Herre 2010; Jandér et al. 2012; Wang et al. 2014b). The active collection and subsequent deposition of pollen nonetheless costs time and energy, and may increase the risk of infection by nematodes and mites (Jauharlina et al. 2012).

Despite the apparent advantages of active pollination for both the insects and their hosts, phylogenetic analyses have revealed that the loss of active pollination behavior has occurred several times among fig wasps (Kjellberg et al. 2001; Cook et al. 2004). Loss of active pollination behavior by the sole pollinator of a *Ficus* species would result in an unstable situation, because the plant could set little, if any, seed, but if the tree is also host to a second species of pollinator, then the scenario can remain stable, and a nonmutualistic ‘cuckoo’ or ‘cheater’ will be present alongside the pollinator. Cheater species reared from figs of *F. altissima* and *F. microcarpa* in Asia appear to be sister species of the trees’ pollinators and may be derived from them (Peng et al. 2008; A. Cruaud, Pers. Comm.). This contrasts with the two more distantly related *Ceratosolen* species that develop in figs of *F. sycomorus* in Africa, where the cheater species is probably the result of a switch in host (Kerdelhue et al. 1999). All three cheater species retain, but fail to use, pollen pockets, so they are clearly derived from species that at one time displayed active pollination behavior.

Some individuals within species of routinely actively pollinating fig wasps do fail to collect any pollen before they disperse from their natal figs (Jandér et al. 2012), and the amount of pollen that individual wasps collect also varies according to the extent that they are competing for access to male flowers, suggesting that lack of pollen collection does not necessarily reflect an adaptive strategy (Kjellberg et al. 2014).

The more efficient pollen collection achieved by active pollinators allows actively pollinated *Ficus* species to have figs that contain far fewer male than female flowers (Kjellberg et al. 2001). Anther‐to‐ovule ratios (A/O ratios) (the size of anthers varies greatly between *Ficus* species, Berg et al. 2005), a coarse indicator of pollen‐to‐ovule ratios (P/O ratios), are therefore a good predictor of pollination mode (Kjellberg et al. 2001; Jousselin et al. 2003; Cook et al. 2004; Wang et al. 2014a). Low A/O ratios (<0.16) are indicative of active pollination mode and higher A/O ratios (more than 0.21) are indicative of passive pollination (Kjellberg et al. 2001).

It has generally been assumed that mode of pollination is a species‐wide phenomenon, with each species of fig tree routinely either actively or passively pollinated (Kjellberg et al. 2001). The occurrence of some outlier figs presenting exceptionally large numbers of male flowers has been documented in one actively pollinated *Ficus* species (Suleman et al. 2013, 2014). However, a systematic survey of A/O ratios across the geographic range of an actively pollinated fig tree presenting locally high A/O ratios has never been attempted. We therefore set out to examine variation in A/O ratios in natural populations of such a *Ficus* species and to ask whether there was geographic structuring in the variation. The loss of active pollination behavior in fig wasp pollinators will favor host plants that produce more pollen. Another route to a change from active to passive pollination can also be envisioned, with selection favoring plants that increase pollen production, while the fig wasps still maintain active pollination.

Using a dioecious Chinese species, *F. tikoua*, we address here the following questions: (1) How large is the range in male flower numbers within male figs of *F. tikoua*, and does the distribution of male flowers within figs change with A/O ratio? (2) Is the variation in male flower numbers continuous or discontinuous? (3) Is there a geographic pattern to the distribution of this floral trait? (4) Are pollinators actively collecting pollen throughout the range of the plant, and are the pollen grains unusually small? and (5) what are the potential drivers for variation in male flower numbers within this species?

## Materials and Methods

### Ficus tikoua *and its pollinator*

*Ficus tikoua* Bur. is a dioecious prostrate shrub with a distribution that extends from north‐eastern India and southern China to Laos and Vietnam (Chang and Wu 1998). *F. tikoua* grows mostly in open woodland and wasteland. *F. tikoua* often has its figs partially buried in the ground, hence its Chinese name of *di‐guo* meaning “fruit in soil”. The figs reach about 10–20 mm in diameter when mature and female figs are brown when ripe, suggesting that terrestrial mammals may be the main seed‐dispersal agents. Each fertile male flower (only present in figs on male trees) has two anthers. There is strong seasonal variation in fig production, and fruiting patterns vary between the sexes (Zhao et al. 2014). Crops vary in size, but are often small. Populations of the plant display genetic differences, suggesting that gene flow between populations is limited, allowing for local founder effects (Chen et al. 2011). Many *F. tikoua* figs abort after failing to be entered by pollinators (Zhao et al. 2014), suggesting that pollinators are often limiting reproduction in this species.

*F. tikoua* was previously assigned to Subgenus *Ficus* (Corner 1965), but was shown to belong to Subgenus *Sycomorus* in a molecular phylogeny of *Ficus* (Cruaud et al. 2012), a conclusion that is consistent with the identity of its pollinator, and also with male flower morphology and disposition (F. Kjellberg Pers. Obs., C.C. Berg *in litterae*). The size of the anthers appears similar to that of other species in subgenus *Sycomorus*. Species in subgenus *Sycomorus* are characterized as always having the male flowers in their figs restricted to the area around the ostiole (Berg et al. 2005).

Based on both morphology and mitochondrial COI genes, the undescribed *Ceratosolen* sp. pollinator of *F. tikoua* comprises one species containing three clades with COI p‐distances separated by far less than average distances between sister species in *Ceratosolen* or between congeneric species of Hymenoptera in general (Hebert et al. 2003; Moe and Weiblen 2010; Y. Chen J‐Y. Deng, R‐H. Fu, unpubl. data). Molecular comparisons suggest that this *Ceratosolen* pollinator is relatively isolated and basal within the genus (Cruaud et al. 2012; J‐Y. Rasplus, Pers. Comm.). *F. tikoua*, section *Hemicardia*, and section *Sycomorus* may form a monophyletic clade within subgenus *Sycomorus* (a possibility suggested by Berg 2004; for *Hemicardia* and *Sycomorus*), and their pollinators seem to form a parallel monophyletic clade within genus *Ceratosolen* (Cruaud et al. 2012).

All fig trees in subgenus *Sycomorus* are pollinated by *Ceratosolen* species, and these fig wasps do not pollinate fig trees in other subgenera. *Ceratosolen* species possess well‐developed pollen pockets and are therefore (with the exception of the African cheater *C. galili*) believed to be active pollinators. To date, there is no reported case of passive pollination within subgenus *Sycomorus*, a feature that sets this subgenus apart.

### Sample sites and sampling methods

Male figs of *F. tikoua* containing fig wasp pupae/adults in their galls and mature or almost‐mature male flowers were collected in May to June 2013 and 2014 from populations separated by more than 1500 Km north–south and 800 Km east–west. The prostrate life‐form of *F. tikoua* makes the identification of individual plants difficult (Zhao et al. 2014). Between one and 13 male figs, depending on availability, were collected from within a maximum area of 1 × 1 m. The minimum distance between sample squares was 30 m and each sample area was regarded as being from a different individual plant. In total, 1044 figs from 206 individuals in 41 populations were sampled (Table 1).

**Table 1:** Locations of sampled *F. tikoua* populations (ordered from N to S). A single female fig wasp was examined for pollen pocket and body surface pollen from each of 38 populations. The fig groups O, I, and S correspond to figs with ostiolar, intermediate, and scattered male flowers, respectively

Location	ID	Latitude (°N)	Longitude (°E)	Altitude (m)	N (plants)	Male flower distribution frequencies (N)			Fig wasps		
						O	I	S	Source fig male flower distribution	Pollen in pollen Pockets?	Nos. of surface pollen grains
Mianyang	SMY	31.34	104.53	479	9	33	0	0	O^a^	Yes	19
Fengya	SFY	30.14	106.90	292	2	19	0	0	O	Yes	/
Chongqing	SCQ	29.40	106.41	308	3	23	0	0	O	Yes	/
Hanyuan	SHY	29.30	102.50	969	4	19	1	0	O^a^	Yes	1
Shimian	SSM	29.16	102.36	1175	10	67	0	0	O^a^	No	0
Naxi	SNX	28.75	105.55	482	4	27	1	0	O	No	/
Guling	SGL	28.05	105.79	595	5	40	6	6	/	/	/
Xichang	SXC	27.83	102.27	1717	5	29	1	0	O^a^	Yes	1
Wengan	GWA	27.15	107.42	1025	4	19	0	0	O	Yes	/
Yanyuan	SYY	27.14	101.52	1574	10	41	0	0	O	No	/
Zhaotong	YZT	27.12	103.43	1452	2	10	0	0	O	No	/
Kaiyang	GKY	27.07	107.06	983	7	21	4	10	O	Yes	/
Ningnan	SNN	27.02	102.77	932	2	20	1	0	O	No	/
Ludian	YLD	27.00	103.32	1493	4	20	0	0	O	No	/
Qiaojia	YQJ	26.90	102.96	1399	7	34	0	0	O^a^	Yes	2
Tianlong	GTL	26.41	106.14	1375	8	9	2	29	S	Yes	/
Panzhihua	SPZ	26.40	101.77	1243	8	73	0	0	O	Yes	/
Yongren	SRZ	26.30	101.67	1905	4	10	0	0	O^a^	No	0
Sandu	GSD	26.05	107.88	477	12	41	6	7	S	Yes	/
Guanling	GGL	25.93	105.64	851	10	0	0	44	S^a^	Yes	40
Sanjiang	GSJ	25.78	109.24	167	8	36	4	0	O^a^	Yes	14
Congjiang	GCJ	25.77	108.88	215	10	35	10	5	I^a^	Yes	61
Wuding	YWD	25.59	102.19	2112	1	0	0	5	S	Yes	/
Nandan	GND	25.18	107.46	761	9	9	2	23	S^a^	Yes	2
Kunming	YKM	25.07	102.61	2241	3	0	0	21	S	Yes	/
Luoping	YLP	25.04	104.53	1248	8	0	0	53	S	Yes	/
Shizong	YSZ	24.79	104.16	1738	3	0	0	5	S	Yes	/
Longlin	GLL	24.79	105.49	435	3	0	0	6	S^a^	Yes	13
Luxi	YLX	24.67	103.56	1901	5	0	7	6	S	No	/
Donglan	GDL	24.57	107.74	322	2	10	0	0	O^a^	Yes	4
Hechi	GHC	24.54	108.49	242	3	4	6	5	S^a^	Yes	12
Luzhai	GLZ	24.53	109.88	130	2	10	0	0	O^a^	Yes	13
Luliang	YLL	24.51	103.59	1917	1	0	0	4	S	No	/
Yizhou	GYZ	24.35	108.76	282	7	12	5	14	/	/	/
Jingyan	YJY	24.21	102.65	1773	1	0	0	3	S	Yes	/
Puzhehei	YPZ	24.09	104.05	1552	3	0	0	13	/	/	/
Tianyang	GTY	23.91	107.05	1703	2	0	0	10	S^a^	Yes	41
Yanshan	YYS	23.84	102.53	390	5	0	0	14	S	Yes	/
Jianshui	YJS	23.64	104.3	1683	2	0	0	5	S	No	/
Mingjiu	YMJ	23.60	103.07	1598	4	0	0	8	S	No	/
Mengzi	YMZ	23.43	103.75	1281	4	0	0	21	S	Yes	/

a Specimens allowed to emerge naturally from figs.

The male figs were kept in fine‐mesh bags to let the fig wasps emerge naturally, or cut open if necessary to release the wasps. No emerging female fig wasps were conspicuously pollen‐coated to the naked eye. Between three and five females that had emerged naturally from 15 male figs (each from a different population) were kept in 0.1 mol/L phosphate buffered glutaraldehyde solution for scanning electron microscopy. All the remaining fig wasps were stored in absolute ethanol. The figs were also kept in alcohol prior to dissection under a stereoscope (Leica EZ4D, Germany). The numbers and position of male flowers (whether concentrated around the ostioles or scattered throughout the figs) were recorded, together with the total number of female flowers.

The ventral surfaces of the mesosoma of 38 female *Ceratosolen* sp. (from 38 populations) were examined using a scanning electron microscope (Zeiss Ultra‐55, Germany) to check for the presence of coxal combs and pollen pockets. Among them, 15 were naturally emerged wasps kept in SEM preserving liquid. The rest had been stored in alcohol with other females. Pollen grain numbers were counted on the bodies outside pollen pockets on scanned views of the 15 wasps that had been stored in phosphate buffered glutaraldehyde solution and prepared for SEM. The sizes of pollen grains on the bodies of eight of the wasps were measured using Photoshop CS6.0. The maximum length and the maximum width (at right angles to maximum length) were recorded for up to 15 pollen grains on each pollinator, depending on how many were clearly visible.

Branches bearing mature male figs or receptive female figs in Mianyang (in the Sichuan Basin) were cut and taken back to the laboratory in June 2015. No appropriate receptive male figs were sampled because male trees produced very few receptive figs at that time. The mature male figs were cut into half and put under a stereoscope (Leica EZ4D) when adult female fig wasps were emerging naturally from their galls and their behaviors were recorded. Naturally emerged females were also introduced into receptive female figs. The receptive figs were then cut into half and put under a stereoscope more than 10 min after fig wasp introduction. The behaviors of the foundresses were again recorded.

### Statistical analyses

Generalized linear models (GLMs) that assumed a quasi‐Poisson distribution of residuals were used to assess the relationships between male flower location (ostiolar, scattered, and intermediate types; intermediate figs were those with most male flowers concentrated around the ostiole, but with a few (<10) scattered elsewhere around the fig cavity), male flower numbers, and floral sex ratios (A/O ratios – each male flower has two anthers). The GLMs were followed by Tukey tests (Tukey HSD). Comparisons of pollen size and the number of visible pollen grains outside the pollen pockets of fig wasps reared from ostiolar and scattered figs were performed also using GLMs. All statistical analyses were performed using R 2.15.0 (R Development Core Team, 2012).

## Results

### Geographic variation in the distribution of male flowers within male figs

**Figure 1:**
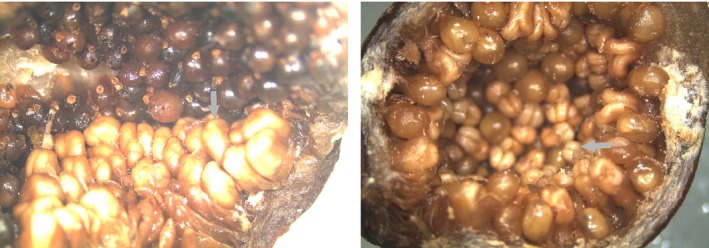
The major distribution patterns of male flowers in the male figs of *Ficus tikoua*: Ostiolar (left) and Scattered (right). Male flowers are highlighted by gray arrows.

Most of the figs had either all their male flowers located around the ostiole or had male flowers scattered more or less evenly throughout the figs (Fig. 1). A small number of intermediate figs were also recorded (56/1044). These had most of their male flowers concentrated around the ostiole, but also had small numbers of male flowers (<10) scattered elsewhere. We refer to them as ostiolar, scattered, and intermediate figs, respectively. Within our samples as a whole (all sites combined), most of the plants (sample squares) had only one type of fig present (102 ostiolar, 64 scattered, and 1 intermediate). A further 39 plants had two or even all three types of figs present (Table S1). Some of these sample areas may have included figs from more than one male plant.

**Figure 2:**
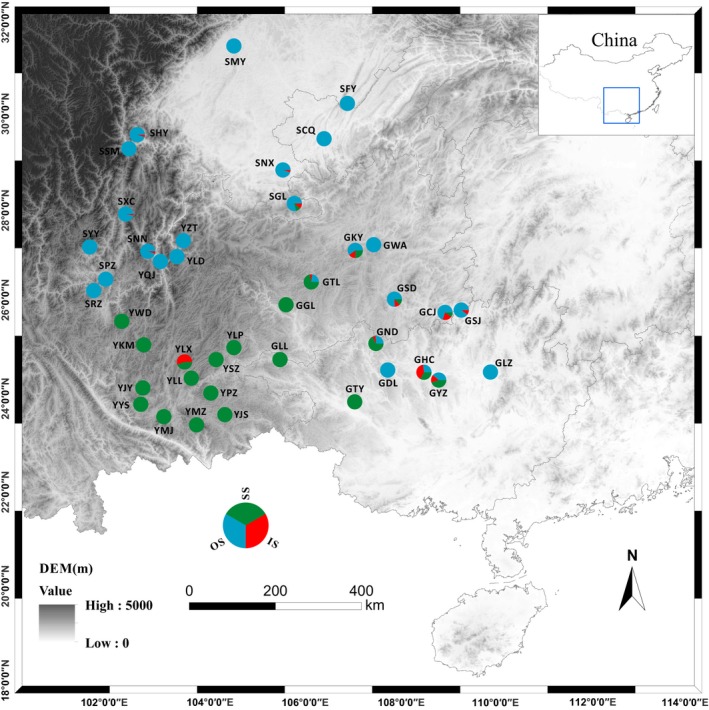
The distributions and frequencies of *Ficus tikoua* figs with ostiolar (blue), intermediate (red), or scattered (green) male flowers in southwest China.

Most populations were monomorphic for male flower location. Thirteen populations had only ostiolar figs, 14 had only scattered figs, and eight populations had all three fig types. Intermediate figs were always found in combination with other male flower distribution types except for one individual in population GHC (Table S1). The two main types (ostiolar vs. scattered) of figs were geographically separated and largely allopatric. Plants with scattered male flowers were concentrated on the Yungui Plateau, and plants with ostiolar figs were mainly recorded in the Sichuan Basin and nearby mountains. Intermediate types were recorded from the more eastern regions (Fig. 2). In the eight populations where a mixture of all three fig types were present, 19 plants had figs with only ostiolar flowers, 14 had figs with only scattered flowers, and 1 plant had figs only with intermediate flowers, while the remaining 27 plants had more than one fig type present (Table S1).

### Flower numbers and A/O ratios in figs of *F. tikoua*

The numbers of female flowers in figs with ostiolar, intermediate, and scattered male flowers were 228.61, 204.05, and 214.67, respectively (Table 2). A significant difference was present in female flower numbers among the three types of figs (GLM, *P *=* *0.003). Female flower numbers were similar in figs with ostiolar and scattered male flowers (Tukey HSD *P *=* *0.583), whereas intermediate figs had significantly fewer female flowers than the other two floral types (Tukey HSDs, *P *=* *0.045 and 0.016 when compared with ostiolar and scattered figs, respectively).

**Table 2:** The average flower numbers and anther‐to‐ovule ratios (A/O ratios) of different fig types

Fig Types	Fig Nos	Female flower		Male flower		A/O ratios	
		Means	SDs	Means	SDs	Means	SDs
Ostiolar	667	228.61	76.49	37.08	8.57	0.35	0.13
Intermediate	56	204.05	67.66	43.77	13.16	0.47	0.20
Scattered	321	214.67	69.48	119.30	65.50	1.32	1.28

Male flower numbers differed much more strongly between the three groups of figs. Figs with an ostiolar distribution of male flowers had the smallest number of male flowers and those with a scattered distribution had the most male flowers. Figs with an intermediate male flower distribution also had intermediate numbers of male flowers (Table 2). Figs with different male flower locations had significantly different numbers of male flowers (GLM, *P *<* *0.001). Tukey tests showed that the numbers of male flowers in scattered figs were significantly higher than those in both ostiolar and intermediate figs (*P *<* *0.001), but there was no significant difference in male flower numbers between ostiolar and intermediate figs (*P *=* *0.397).

**Figure 3:**
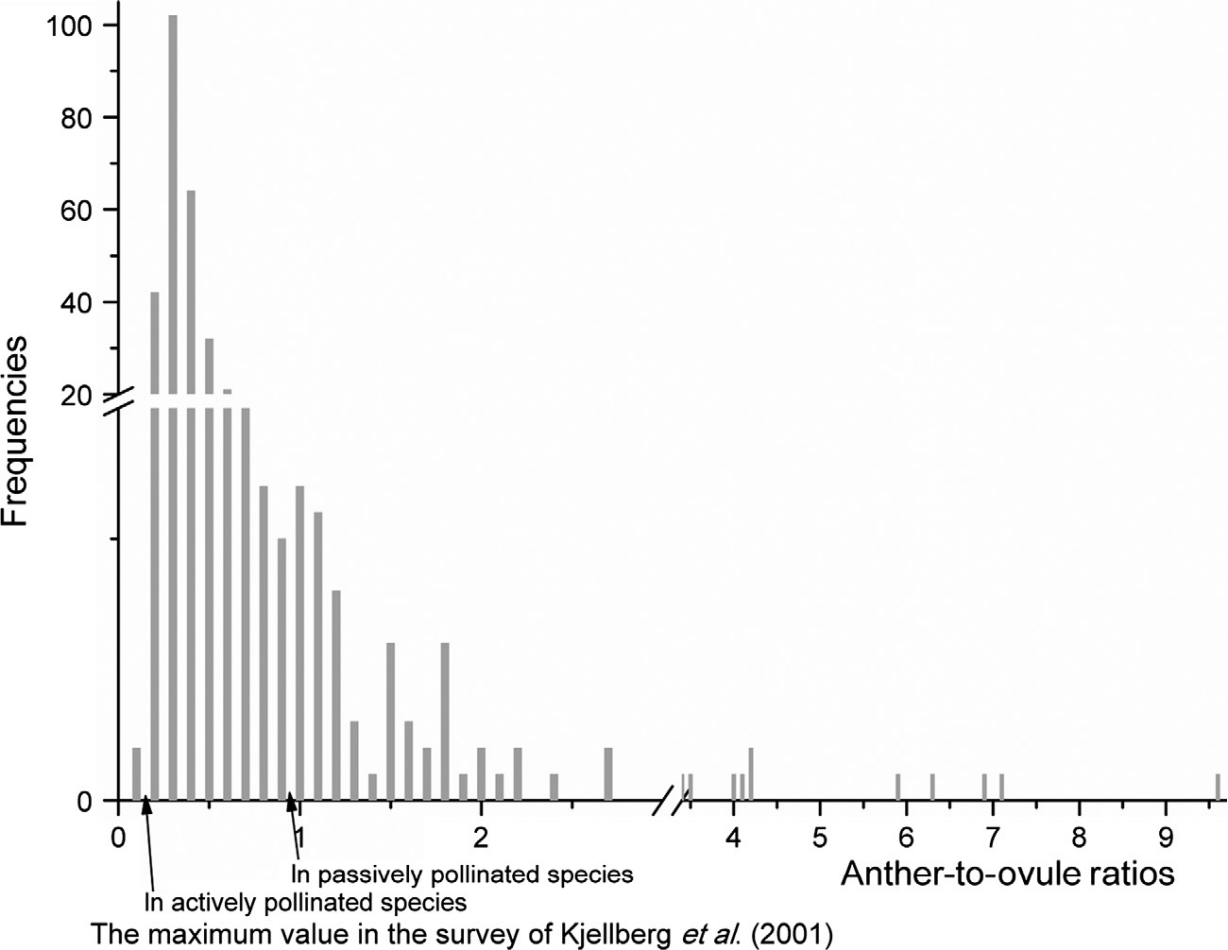
Anther‐to‐ovule ratios in male *Ficus tikoua* figs.

Each male flower in figs of *F. tikoua* contains two anthers and each female flower has one ovule. Across the figs of *F. tikoua* as a whole, A/O ratios averaged 0.700 ± 0.842 (*n* = 1044 figs), but the large variation in male flower numbers resulted in exceptional intraspecific variation in A/O ratios (Table S2). A/O ratios within individual figs ranged from 0.096 (about one anther for 10 female flowers) to 10.0 (10 anthers for each female flower). Only 2% of the figs fell within the range described by Kjellberg et al. (2001) as being typical of actively pollinated figs (A/O < 0.16, 7/1044), leaving 98% of the figs showing A/O ratios that have been considered indicative of passively pollinated *Ficus* species (A/O ≥ 0.21, 1022/1044) (Fig. 3), and in some cases, the A/O ratios far exceeded those considered typical of even passively pollinated species.

Anther‐to‐ovule (A/O) ratios in figs with scattered male flowers were 1.32, compared with 0.35 in figs with ostiolar flowers and 0.47 in figs with an intermediate male flower distribution (Table 2). A/O ratios in the three groups were significantly different (GLM, *P *<* *0.001). Figs with scattered male figs had significantly higher A/O ratios than ostiolar or intermediate figs (*P *<* *0.001, Tukey tests), but no significant differences between ostiolar and intermediate figs were present (*P *=* *0.462). This pattern remained when only figs in the populations where all three fig types were coexisting were included. Scattered figs again had significantly higher male flower numbers and A/O ratios than ostiolar and intermediate figs in seven out of eight population (*P *<* *0.05). Population SGL was the exception. No significant differences in female flower numbers were detected between the three types of figs in most populations, but population GHC was an exception (Table 3). Pairwise assessments of between‐population variation in male, female flower numbers and A/O ratios within the three groups of figs (ostiolar, intermediate, and scattered) failed to find significant differences in most cases (Tukey HSD tests, data not shown).

**Table 3:** The results of Tukey's HSD tests (*P* values) comparing flower numbers and floral ratios in male figs with different male flower distribution patterns. Only populations where all three groups of figs were present are included. Significant values are in bold

Population	No. of figs			Male flower Nos.			Female flower Nos.			A/O ratio		
	O	I	S	O‐I	S‐I	S‐O	O‐I	S‐I	S‐O	O‐I	S‐I	S‐O
GHC	4	6	5	0.929	**0.001**	**<0.001**	**0.001**	**0.004**	0.885	0.374	**0.001**	**0.004**
GYZ	12	5	14	0.430	**<0.001**	**<0.001**	0.882	0.841	0.998	0.817	**0.000**	**0.024**
GND	9	2	23	0.715	**<0.001**	**<0.001**	0.342	0.991	0.694	0.582	**0.002**	**0.033**
SGL	40	6	6	0.888	0.791	0.989	1.000	0.910	0.949	0.479	0.862	0.876
GKY	21	4	10	0.419	**<0.001**	**<0.001**	0.931	0.201	0.701	0.375	**<0.001**	**0.032**
GTL	9	21	29	0.992	**0.001**	0.165	0.962	0.984	0.926	0.988	**0.026**	0.426
GSD	41	6	7	0.689	**<0.001**	**<0.001**	0.137	0.083	0.996	0.872	**0.000**	**0.001**
GCJ	35	10	5	0.673	**<0.001**	**<0.001**	0.334	0.991	0.689	0.542	**0.001**	**0.032**

### The pollination mode of the Ceratosolen pollinator of *F. tikoua*

Active pollination was confirmed by direct observation of fig wasp behavior in both the mature male figs (*n* = 15) and receptive female figs (*n* = 10) collected in Mianyang (located within an area where most figs have an ostiolar male flower distribution). The adult female fig wasps emerging from galls competed for appropriate anthers, and collected pollen actively in mature male figs (Video S1). When introduced into receptive female figs, they deposited pollen onto the stigmas when they were ovipositing (Video S2).

**Figure 4:**
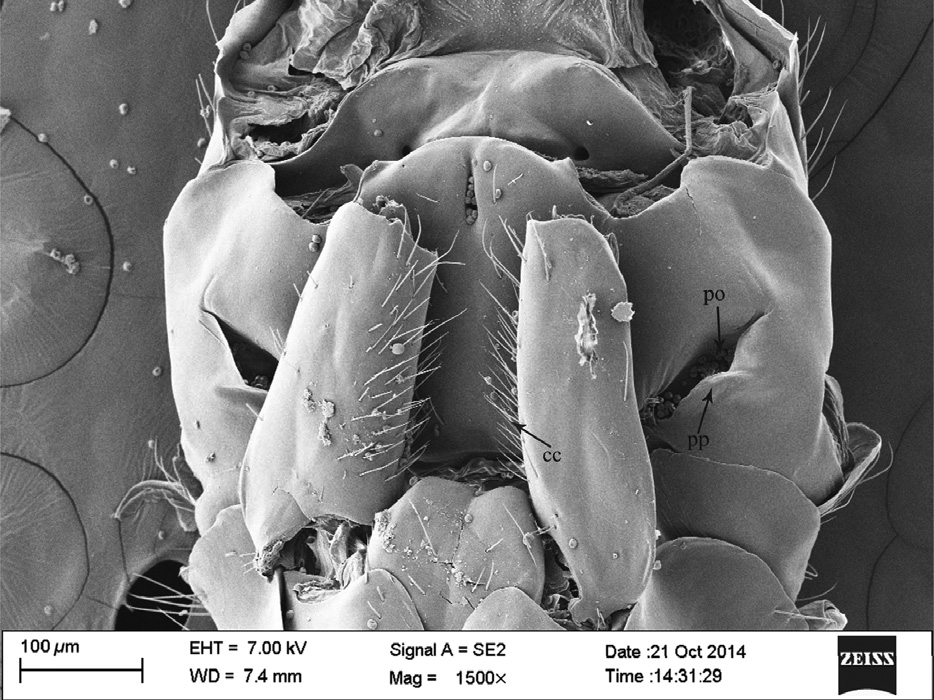
SEM view of ventral mesosoma of a female *Ceratosolen* sp. from *Ficus tikoua*. pp, pollen pocket; po, pollen; cc, coxal comb.

Active pollination was also supported by the SEM images of the undersides of 38 female pollinators from 38 populations (Table 1). The scanned fig wasps were reared from 1 intermediate, 19 ostiolar, and 18 scattered figs. Well‐developed pollen pockets and coxal combs were present in every specimen, irrespective of the male flower distribution patterns in their natal figs (Fig. 4). Among the 15 ‘naturally emerged’ females (their natal figs had nonetheless been removed from the trees before they emerged), pollen was present in the pollen pockets of 13 individuals. The other two females had no visible pollen present anywhere. They had emerged from ostiolar figs. Pollen was also present elsewhere on the bodies (other than in their pollen pockets) of most of the 13 females that had loaded their pollen pockets. Visible pollen grains outside pollen pockets ranged from 1 to 19 and 2 to 41 on females reared from 7 ostiolar and 5 scattered figs, respectively (the two pollen‐free fig wasps were excluded). Females emerging from figs with ostiolar male flowers had 7.71 ± 7.42 pollen grains (mean ± SD) visible on their bodies (away from their pollen pockets), compared with 21.60 ± 17.78 pollen grains visible on pollinators reared from figs with scattered male flowers. The difference was significant (GLM, *P *=* *0.044). However, we had not scanned the whole bodies of the females and only included a small number of fig wasps from each natal fig type, so more data are needed to confirm this pattern.

Female flowers in receptive female figs of *F. tikoua* presented tubular stigmas sticking together and forming a synstigma, whereas female flowers in male figs presented well‐separated stigmas (Fig. S1). The anthers did not dehisce spontaneously in most figs that were opened before the fig wasps emerged from their galls. However, a few figs in Mianyang and Qujing had anthers that had dehisced when all the fig wasps were still in their natal galls (Fig. S2).

### Pollen grain size in *F. tikoua*

The pollen of *F. tikoua* on the bodies of the *Ceratosolen* sp. females was ellipsoidal in shape, 8.51 ± 0.83 *μ*m long, and 7.10 ± 1.14 *μ*m wide (mean ± SD, *n* = 117 pollen grains from eight fig wasps) (Table 4). There was no difference in the size of the pollen from fig wasps that emerged from figs with ostiolar (*n* = 4) and scattered (*n* = 4) male flowers (GLM, *P *=* *0.318 for length, *P *=* *0.919 for width).

**Table 4:** The size of *Ficus tikoua* pollen carried by *Ceratosolen* sp. females

Source population	Fig types of Natal Figs	N pollen grains	Pollen size (um)			
			Major axis		Minor axis	
			Mean	SD	Mean	SD
GDL	Ostiolar	12	8.85	0.75	7.15	0.47
GLZ	Ostiolar	15	8.76	0.85	7.00	0.65
SMY	Ostiolar	15	8.12	0.86	6.80	1.85
GSJ	Ostiolar	15	8.66	0.64	7.50	0.44
GHC	Scattered	15	8.15	0.96	6.28	1.11
GGL	Scattered	15	8.47	0.78	7.25	1.42
GLL	Scattered	15	8.91	0.75	7.08	1.61
GTY	Scattered	15	8.20	0.60	7.74	0.57
Mean		14.63	8.51	0.83	7.10	1.14

## Discussion

Direct observations and video images show that *Ceratosolen* sp. in Mianyang displays active pollination behavior. In agreement, all SEM‐scanned wasps collected from 38 populations possessed well‐developed pollen pockets and coxal combs. Moreover, pollen was observed to be present in the pollen pockets throughout the investigated range, confirming that the fig wasps do actively load pollen into their pockets (Fig. 4). Morphologies of the stigmas in receptive figs and anthers in most mature male figs of *F. tikoua* corresponded to those typically observed in actively pollinated dioecious figs and were strikingly different from those observed in passively pollinated figs (Jousselin and Kjellberg 2001). The female flowers in receptive female figs of *F. tikoua* have tubular stigmas that stick together to form a synstigma that allows secondary dispersal of pollen through lateral pollen tube growth. In contrast, female flowers in receptive male figs presented well‐separated stigmas, which allow precise deposition of pollen onto the stigma of each flower into which a female is ovipositing (Jousselin and Kjellberg 2001) (Fig. S1). In addition, the anthers had not dehisced spontaneously in most of the figs that had been opened just before the fig wasps emerged from their natal galls, a trait typical of actively pollinated *Ficus* species (Galil and Meiri 1981). Hence, this suite of traits in both *F. tikoua* and its pollinator is consistent with typical active pollination.

However, while floral ratios (A/O ratios) are strong indicators of pollination mode in *Ficus* species, and are typically consistent within species (Kjellberg et al. 2001; Cook et al. 2004), male figs of *F. tikoua* exhibit large variation in the numbers of male flowers that they contain. This resulted in exceptional intraspecific variation in A/O ratios that ranged from about 1:10 to 10:1 in figs collected from different plants. Large variation in floral ratios has previously been reported in figs of *F. montana*, an actively pollinated species, but those data were based on an experimental population where a limited number of atypical individuals had high numbers of anthers and it remains to be confirmed whether similarly large variation occurs in natural populations of that species (Suleman et al. 2014). The floral ratios in a minority of *F. tikoua* figs were suggestive of active pollination, but the figs on most plants were typical of a passively pollinated species (Kjellberg et al. 2001), and some figs had floral ratios that are higher than those recorded previously for any *Ficus* species.

In *Ficus*, the male plant has to allocate resources between producing pollen vectors (fig wasps) and producing pollen. The higher ratios seen in passively pollinated compared with actively pollinated *Ficus* species are a reflection of a contrast in relative efficiency of pollen loading onto the fig wasps and their subsequent deposition of pollen onto the stigmas. Therefore, the high floral ratios recorded for most *F. tikoua* figs suggest that the pollen that is actively loaded by the fig wasps into their pollen pockets and subsequently deposited in female figs may be insufficient for efficient seed set in this species. Actively pollinating foundresses are thought to deliberately fertilize the ovules in which they oviposit because it increases larval survivorship (Jandér et al. 2012). In dioecious figs, pollen deposition is extremely precise, as almost exclusively it is the stigmas of flowers that have received an egg that are pollinated. (Jousselin and Kjellberg 2001). Natural selection should favor a match between the number of pollen grains that each foundress collects and the average number of eggs she may deposit, given that foundresses have some control over which stigmas they deposit pollen on and also how many pollen grains they deposit per stigma. Such a match was revealed in an actively pollinated species, *F. condensa*. Its pollinators each carried an average of 884 pollen grains, while the male figs of this species, into which the pollinators lay their eggs, had an average of 833 female flowers (F. Kjellberg, unpubl. data). The numbers of female flowers in male figs of *F. tikoua* (averaging around 230) were relatively consistent among figs irrespective of male flower numbers. Assuming egg loads equal to or smaller than the numbers of female flowers in a single fig, each *Ceratosolen* sp. foundress might therefore be expected to attempt to load her pockets with sufficient pollen grains to fertilize all of the 230 female flowers where she can potentially oviposit. In practice, adult offspring numbers were found to be slightly lower than the number of female flowers available in male figs collected in Mianyang (205 ± 44 offspring per fig in spring crops, 193 ± 53 in summer crops Zhao et al. 2014). The optimum number of pollen grains carried by a fig wasp to maximize its own reproductive success if it enters a male fig is, however, not necessarily the same as the number that would maximize seed set in a female fig and hence the male reproductive success of its natal host plant if the fig wasp enters a fig on a female plant. Indeed, far more female flowers are present in female figs of *F. tikoua* than in male figs and far more seeds are produced per female fig than fig wasp offspring per male fig (782.0 ± 111.6 seeds; Zhao et al. 2014). Consequently, the quantity of pollen a foundress is selected to load into her pockets is probably not sufficient to ensure full seed set in female figs and additional foundresses may be needed to enter each female fig to achieve full seed sets. However, pollinator shortages in *F. tikoua* may mean that optimal foundress numbers for female figs may rarely be achieved. In this situation, selection should favor any male plants that can increase the numbers of pollen grains carried by the fig wasps that emerge from their figs even when this results in the fig wasps carrying more pollen than they themselves require to maximize their own reproductive success. The additional pollen grains carried on the bodies of *Ceratosolen* sp., may therefore be beneficial to the plant, if it results in more seeds developing in female figs. No change in pollinator behavior appears to be required to transport pollen on the body surfaces of these insects (Galil and Neeman 1977) and ‘cheater’ fig wasps associated with the actively pollinated *F. sycomorus* and *F. microcarpa* can generate small numbers of seeds in figs they enter, despite failing to actively collect pollen (Compton et al. 1991; Wang et al. 2015).

An increase in the numbers of male flowers in *F. tikoua* figs is associated with a switch from the ostiolar disposition reported from all other species from subgenus *Sycomorus* (Berg et al. 2005), all of which are believed to be actively pollinated, to a scattered distribution. Shifts from ostiolar to scattered distribution of male flowers seem to have taken place in a limited number of *Ficus* sections. In the case of section *Urostigma*, scattered distributions of male flowers have been reported for the figs of *F. densifolia* and *F. prolixa*, two species where putative reversals to passive pollination have taken place (Kjellberg et al. 2001). Most *F. tikoua* individuals had figs with just one of the forms of male flower distribution and we cannot be sure that the apparent exceptions were not the result of our sampling of figs from two or more intergrowing male plants.

There were clear differences in the geographic distributions of plants with scattered versus ostiolar male flowers, with the former concentrated in populations from the south and west of the sample area, on the Yungui Plateau (Fig. 2). What could be the driver of this regional difference? Cruden (1977, 2000) suggested that intraspecific variation in P/O ratios may be linked to different pollinator species, but molecular data suggest that *F. tikoua* has a single pollinator species throughout our sampled regions (Y. Chen, J‐Y. Deng, R‐H. Fu, unpubl. data). More surface pollen grains were attached to the bodies of female *Ceratosolen* sp. reared from the figs with greater numbers of male flowers, where they are scattered around the interior, rather than concentrated near the ostiole (the area through which the fig wasp adult offspring emerge and escape). The region in the southwest of our sampling area where male flower numbers are higher is an elevated plateau, and lower temperatures may be impacting on plant phenology and the survivorship of the fig wasps, together with the vigor of foundresses trying to oviposit within the figs. Pollen limitation may therefore be particularly acute for *F. tikoua* in this region. Alternatively, the current situation may be transitory, with plants that produce many male flowers having originated in that area and currently expanding their range. Further studies will be required to answer these questions.

Within a context of apparently limiting pollen transfer, the production of more, smaller sized pollen grains should be favored. Indeed sex allocation theory predicts that pollen–ovule ratios are correlated with pollen grain size (Gotzenberger et al. 2007). However, the size of *Ficus* pollen grains in general is already very small (about 10 micrometers) and the size of the pollen of *F. tikoua* is typical of that seen in other *Ficus* species (Wang et al. 2014a). Hence, evolving smaller pollen size is probably not an option in *Ficus*. High floral ratios in a male fig are an alternative way to make more pollen available for each potential pollen vector or to load passively additional pollen on each potential pollen vector, but this is achieved at the expense of producing fewer vectors, because resources and space for male flowers are necessarily diverted from the female flowers inside which pollinator larvae develop. Floral ratios in male figs therefore reflect a compromise between the numbers of vectors generated and the amount of pollen carried by each vector, as pollen loading conditions in natal figs appear to impact the future seed set capacity of both active and passive pollinators (Kjellberg et al. 2014). Hence, the high floral ratios in *F. tikoua* could result from selection on the fig tree to deposit additional quantities of pollen on the bodies of female wasps, to add to the pollen that the fig wasps are actively collecting, in order to increase seed set in female figs.

Although the pollinator of *F. tikoua* effectively loads pollen in its pockets and presents pollen deposition behavior, at least in parts of its range, it presents one scenario in which active pollination could eventually be lost in dioecious *Ficus* species, an event that has occurred several times in the history of the relationship between figs and fig wasps (Jousselin et al. 2003). Given the large amounts of pollen being produced by some *F. tikoua* figs, any individuals of *Ceratosolen* sp. that fail to fill their pollen pockets, and no longer actively pollinate, can still be transporting pollen on their body surface and thereby pollinate passively. If there is a significant cost to active pollination, then such individuals will be favored. The current pollination mode in *F. tikoua* may therefore be inherently unstable. The anthers of a few figs sampled in both the Sichuan Basin (Mianyang) and Plateau (Qujing) dehisced spontaneously, a feature characteristic of passively pollinated fig species. This might indicate that the pollination mode in *F. tikoua* is unstable.

## Conflict of Interest

None declared.

## Supporting information


**Table S1.** Comparisons of the numbers of sample squares (equivalent to individual plants) with different fig male flower distributions in the sampled populations of *Ficus tikoua*.
**Table S2.** The flower numbers and anther‐to‐ovule (A/O) ratios in figs of *Ficus tikoua* from southwest China.
**Fig. S1.** The interiors of female figs of *Ficus tikoua* (up), *F. hainanensis* (middle), and *F. carica* (down) at the receptive stage when pollinators enter.
**Fig. S2.** The interiors of male figs collected in Mianyang (up) and Qujing (down), showing anther dehisced spontaneously before wasp emerging from the gall.

**Video S1.** The behaviors of female pollinating fig wasps in mature male fig of *Ficus tikoua*.

**Video S2.** The behaviors of female pollinating fig wasps in receptive female fig of *Ficus tikoua*.
