# Supplementary material for: Extremely high proportions of male flowers and geographic variation in floral ratios within male figs of Ficus tikoua despite pollinators displaying active pollen collection
Source: Ecol Evol. 2016 Jan 9;6(2):607–19. doi: 10.1002/ece3.1926 (PMC4729252; doi:10.1002/ece3.1926)

Table S1 Comparisons of the numbers of sample squares (equivalent to individual plants) with different fig male flower distributions in the sampled populations of *Ficus tikoua*. O, I, and S indicate ostiolar, intermediate and scattered male flowers respectively. The population abbreviations are explained in table 1. The eight populations with all three fig types coexisting are highlighted in bold.

| POP | ALL | O | O+I | O+S | I | I+S | O+I+S | S |
| --- | --- | --- | --- | --- | --- | --- | --- | --- |
| SMY | 9 | 9 | 0 | 0 | 0 | 0 | 0 | 0 |
| SFY | 2 | 2 | 0 | 0 | 0 | 0 | 0 | 0 |
| SCQ | 3 | 3 | 0 | 0 | 0 | 0 | 0 | 0 |
| SHY | 4 | 3 | 1 | 0 | 0 | 0 | 0 | 0 |
| SSM | 10 | 10 | 0 | 0 | 0 | 0 | 0 | 0 |
| SNX | 4 | 3 | 1 | 0 | 0 | 0 | 0 | 0 |
| **SGL** | 5 | 1 | 3 | 1 | 0 | 0 | 0 | 0 |
| SXC | 5 | 4 | 1 | 0 | 0 | 0 | 0 | 0 |
| GWA | 4 | 4 | 0 | 0 | 0 | 0 | 0 | 0 |
| SYY | 10 | 10 | 0 | 0 | 0 | 0 | 0 | 0 |
| YZT | 2 | 2 | 0 | 0 | 0 | 0 | 0 | 0 |
| **GKY** | 7 | 3 | 0 | 1 | 0 | 1 | 1 | 1 |
| SNN | 2 | 1 | 1 | 0 | 0 | 0 | 0 | 0 |
| YLD | 4 | 4 | 0 | 0 | 0 | 0 | 0 | 0 |
| YQJ | 7 | 7 | 0 | 0 | 0 | 0 | 0 | 0 |
| **GTL** | 8 | 1 | 0 | 0 | 0 | 1 | 1 | 5 |
| SPZ | 8 | 8 | 0 | 0 | 0 | 0 | 0 | 0 |
| SRZ | 4 | 4 | 0 | 0 | 0 | 0 | 0 | 0 |
| **GSD** | 12 | 6 | 2 | 1 | 0 | 0 | 2 | 1 |
| GGL | 10 | 0 | 0 | 0 | 0 | 0 | 0 | 10 |
| GSJ | 8 | 5 | 3 | 0 | 0 | 0 | 0 | 0 |
| **GCJ** | 10 | 3 | 3 | 1 | 0 | 0 | 3 |  |
| YWD | 1 | 0 | 0 | 0 | 0 | 0 | 0 | 1 |
| **GND** | 9 | 2 | 1 | 2 | 0 | 0 | 0 | 4 |
| YKM | 3 | 0 | 0 | 0 | 0 | 0 | 0 | 3 |
| YLP | 8 | 0 | 0 | 0 | 0 | 0 | 0 | 8 |
| YSZ | 3 | 0 | 0 | 0 | 0 | 0 | 0 | 3 |
| GLL | 3 | 0 | 0 | 0 | 0 | 0 | 0 | 3 |
| YLX | 5 | 0 | 0 | 0 | 0 | 5 | 0 | 0 |
| GDL | 2 | 2 | 0 | 0 | 0 | 0 | 0 | 0 |
| **GHC** | 3 |  | 1 | 0 | 1 | 0 | 0 | 1 |
| GLZ | 2 | 2 | 0 | 0 | 0 | 0 | 0 | 0 |
| YLL | 1 | 0 | 0 | 0 | 0 | 0 | 0 | 1 |
| **GYZ** | 7 | 3 | 1 | 0 | 0 | 1 | 0 | 2 |
| YJY | 1 | 0 | 0 | 0 | 0 | 0 | 0 | 1 |
| YPZ | 3 | 0 | 0 | 0 | 0 | 0 | 0 | 3 |
| GTY | 2 | 0 | 0 | 0 | 0 | 0 | 0 | 2 |
| YYS | 5 | 0 | 0 | 0 | 0 | 0 | 0 | 5 |
| YJS | 2 | 0 | 0 | 0 | 0 | 0 | 0 | 2 |
| YMJ | 4 | 0 | 0 | 0 | 0 | 0 | 0 | 4 |
| YMZ | 4 | 0 | 0 | 0 | 0 | 0 | 0 | 4 |
| TOTAL | 206 | 102 | 18 | 6 | 1 | 8 | 7 | 64 |

Table S2 The flower numbers and anther-to-ovule (A/O) ratios in figs of *Ficus tikoua* from SW China.

| Pop. | Male Flowers | | | |  | Female Flowers | | | |  | Total Flowers | | | |  | A/O Ratios | | | |
| --- | --- | --- | --- | --- | --- | --- | --- | --- | --- | --- | --- | --- | --- | --- | --- | --- | --- | --- | --- |
|  | Mean | SD | Min | Max |  | Mean | SD | Min. | Max. |  | Mean | SD | Min. | Max. |  | Mean | SD | Min. | Max. |
| SMY | 29.97 | 3.92 | 22 | 39 |  | 192.52 | 57.50 | 100 | 294 |  | 222.48 | 58.09 | 128 | 324 |  | 0.34 | 0.10 | 0.15 | 0.56 |
| SFY | 42.05 | 5.24 | 35 | 59 |  | 247.16 | 35.26 | 168 | 308 |  | 289.21 | 35.67 | 212 | 352 |  | 0.35 | 0.07 | 0.25 | 0.52 |
| SCQ | 33.09 | 7.45 | 21 | 50 |  | 175.78 | 52.47 | 101 | 264 |  | 208.87 | 56.68 | 127 | 300 |  | 0.40 | 0.10 | 0.20 | 0.55 |
| SHY | 38.15 | 5.90 | 28 | 53 |  | 250.60 | 36.63 | 205 | 328 |  | 288.75 | 37.17 | 240 | 368 |  | 0.31 | 0.07 | 0.21 | 0.51 |
| SSM | 35.51 | 4.98 | 25 | 47 |  | 217.30 | 57.48 | 66 | 358 |  | 252.81 | 60.07 | 94 | 399 |  | 0.35 | 0.11 | 0.22 | 0.85 |
| SNX | 45.82 | 11.56 | 32 | 84 |  | 255.96 | 80.45 | 130 | 525 |  | 301.79 | 84.77 | 162 | 578 |  | 0.38 | 0.12 | 0.20 | 0.71 |
| SGL | 44.60 | 13.89 | 27 | 103 |  | 216.71 | 51.47 | 112 | 320 |  | 261.31 | 54.61 | 164 | 380 |  | 0.44 | 0.18 | 0.24 | 1.02 |
| SXC | 33.70 | 5.09 | 21 | 43 |  | 216.03 | 49.54 | 85 | 301 |  | 249.73 | 50.85 | 125 | 336 |  | 0.34 | 0.14 | 0.21 | 0.94 |
| GWA | 37.16 | 9.20 | 23 | 55 |  | 188.63 | 60.95 | 46 | 328 |  | 225.79 | 62.80 | 95 | 369 |  | 0.48 | 0.41 | 0.21 | 2.13 |
| SYY | 40.17 | 6.65 | 24 | 58 |  | 277.63 | 82.47 | 173 | 675 |  | 317.80 | 85.15 | 206 | 725 |  | 0.30 | 0.07 | 0.15 | 0.48 |
| YZT | 32.80 | 4.69 | 27 | 41 |  | 215.00 | 57.16 | 137 | 303 |  | 247.80 | 60.62 | 164 | 344 |  | 0.32 | 0.06 | 0.23 | 0.41 |
| GKY | 52.91 | 29.78 | 23 | 159 |  | 211.26 | 67.13 | 78 | 320 |  | 264.17 | 65.79 | 125 | 362 |  | 0.60 | 0.49 | 0.21 | 2.18 |
| SNN | 38.81 | 7.81 | 29 | 64 |  | 264.71 | 101.61 | 147 | 512 |  | 303.52 | 107.80 | 177 | 576 |  | 0.32 | 0.09 | 0.19 | 0.57 |
| YLD | 37.55 | 9.17 | 26 | 62 |  | 235.95 | 77.45 | 123 | 350 |  | 273.50 | 82.53 | 151 | 393 |  | 0.34 | 0.09 | 0.21 | 0.50 |
| YQJ | 37.15 | 6.63 | 10 | 49 |  | 250.12 | 64.34 | 86 | 350 |  | 287.26 | 67.40 | 124 | 393 |  | 0.32 | 0.12 | 0.10 | 0.88 |
| GTL | 65.08 | 31.07 | 26 | 148 |  | 199.70 | 56.40 | 98 | 333 |  | 264.78 | 65.44 | 130 | 402 |  | 0.70 | 0.42 | 0.25 | 2.31 |
| SPZ | 40.45 | 9.15 | 26 | 66 |  | 293.53 | 114.14 | 102 | 582 |  | 333.99 | 120.02 | 139 | 646 |  | 0.30 | 0.10 | 0.15 | 0.73 |
| SRZ | 37.50 | 7.12 | 28 | 51 |  | 196.90 | 24.04 | 157 | 237 |  | 234.40 | 29.45 | 191 | 288 |  | 0.38 | 0.05 | 0.32 | 0.47 |
| GSD | 44.91 | 22.74 | 21 | 153 |  | 199.80 | 55.16 | 88 | 345 |  | 244.70 | 67.78 | 130 | 463 |  | 0.46 | 0.20 | 0.23 | 1.19 |
| GGL | 126.18 | 56.64 | 34 | 237 |  | 220.25 | 65.85 | 76 | 384 |  | 346.43 | 99.25 | 195 | 621 |  | 1.24 | 0.82 | 0.32 | 5.13 |
| GSJ | 32.43 | 7.17 | 19 | 50 |  | 196.03 | 42.81 | 114 | 280 |  | 228.45 | 42.49 | 147 | 307 |  | 0.35 | 0.12 | 0.19 | 0.61 |
| GCJ | 40.16 | 16.15 | 20 | 110 |  | 173.54 | 59.03 | 58 | 354 |  | 213.70 | 63.81 | 93 | 398 |  | 0.52 | 0.25 | 0.19 | 1.21 |
| YWD | 40.40 | 10.38 | 30 | 53 |  | 148.60 | 68.92 | 41 | 215 |  | 189.00 | 74.06 | 71 | 246 |  | 0.70 | 0.45 | 0.29 | 1.46 |
| GND | 75.68 | 41.87 | 19 | 192 |  | 176.32 | 55.18 | 61 | 277 |  | 252.00 | 55.52 | 133 | 358 |  | 1.08 | 1.15 | 0.23 | 6.30 |
| YKM | 113.33 | 46.73 | 23 | 248 |  | 274.90 | 61.02 | 187 | 480 |  | 388.24 | 81.57 | 266 | 622 |  | 0.85 | 0.41 | 0.19 | 2.20 |
| YLP | 149.21 | 63.69 | 49 | 320 |  | 246.81 | 79.32 | 70 | 431 |  | 396.02 | 100.06 | 143 | 654 |  | 1.36 | 0.87 | 0.44 | 4.96 |
| YSZ | 157.20 | 21.25 | 128 | 181 |  | 228.80 | 60.07 | 147 | 312 |  | 386.00 | 63.65 | 294 | 468 |  | 1.46 | 0.43 | 1.00 | 2.00 |
| GLL | 150.50 | 64.78 | 47 | 213 |  | 234.67 | 81.99 | 122 | 334 |  | 385.17 | 82.84 | 294 | 528 |  | 1.54 | 1.09 | 0.30 | 3.49 |
| YLX | 64.15 | 16.38 | 33 | 79 |  | 277.31 | 60.29 | 189 | 412 |  | 341.46 | 66.50 | 222 | 473 |  | 0.47 | 0.14 | 0.26 | 0.69 |
| GDL | 29.00 | 3.23 | 24 | 34 |  | 235.30 | 30.31 | 189 | 280 |  | 264.30 | 29.81 | 213 | 306 |  | 0.25 | 0.05 | 0.19 | 0.32 |
| GHC | 40.53 | 10.66 | 27 | 63 |  | 192.07 | 28.53 | 162 | 259 |  | 232.60 | 26.71 | 198 | 286 |  | 0.43 | 0.14 | 0.21 | 0.74 |
| GLZ | 31.30 | 6.82 | 20 | 39 |  | 254.90 | 58.37 | 177 | 348 |  | 286.20 | 56.03 | 215 | 372 |  | 0.26 | 0.10 | 0.13 | 0.43 |
| YLL | 115.00 | 32.53 | 90 | 162 |  | 274.00 | 43.52 | 225 | 315 |  | 389.00 | 46.32 | 322 | 426 |  | 0.86 | 0.31 | 0.59 | 1.30 |
| GYZ | 67.61 | 39.03 | 23 | 140 |  | 209.97 | 59.40 | 44 | 346 |  | 277.58 | 73.26 | 120 | 472 |  | 0.75 | 0.67 | 0.19 | 3.45 |
| YJY | 103.67 | 11.24 | 94 | 116 |  | 210.67 | 39.50 | 166 | 241 |  | 314.33 | 45.18 | 267 | 357 |  | 1.01 | 0.19 | 0.84 | 1.22 |
| YPZ | 90.23 | 21.17 | 38 | 114 |  | 242.69 | 35.08 | 198 | 318 |  | 332.92 | 29.75 | 299 | 400 |  | 0.77 | 0.23 | 0.24 | 1.07 |
| GTY | 75.50 | 22.79 | 43 | 118 |  | 171.20 | 23.82 | 110 | 192 |  | 246.70 | 39.79 | 174 | 310 |  | 0.89 | 0.24 | 0.52 | 1.23 |
| YYS | 85.79 | 14.36 | 66 | 112 |  | 217.50 | 40.58 | 153 | 277 |  | 303.29 | 46.41 | 222 | 361 |  | 0.81 | 0.16 | 0.53 | 1.09 |
| YJS | 99.00 | 19.05 | 78 | 122 |  | 227.40 | 25.22 | 190 | 261 |  | 326.40 | 35.70 | 288 | 375 |  | 0.88 | 0.17 | 0.69 | 1.07 |
| YMJ | 171.50 | 23.73 | 138 | 212 |  | 201.88 | 48.78 | 153 | 312 |  | 373.38 | 56.91 | 291 | 478 |  | 1.76 | 0.37 | 1.06 | 2.44 |
| YMZ | 265.48 | 50.41 | 175 | 345 |  | 143.38 | 73.60 | 60 | 301 |  | 408.86 | 100.63 | 251 | 589 |  | 4.61 | 2.33 | 1.91 | 10.00 |

Fig. S1 The interiors of female figs of *Ficus tikoua* (up), *F. hainanensis* (middle) and *F. carica* (down) at the receptive stage when pollinators enter. *F. hainanensis* is an actively-pollinated species, while *F. carica* is a passively-pollinated one.


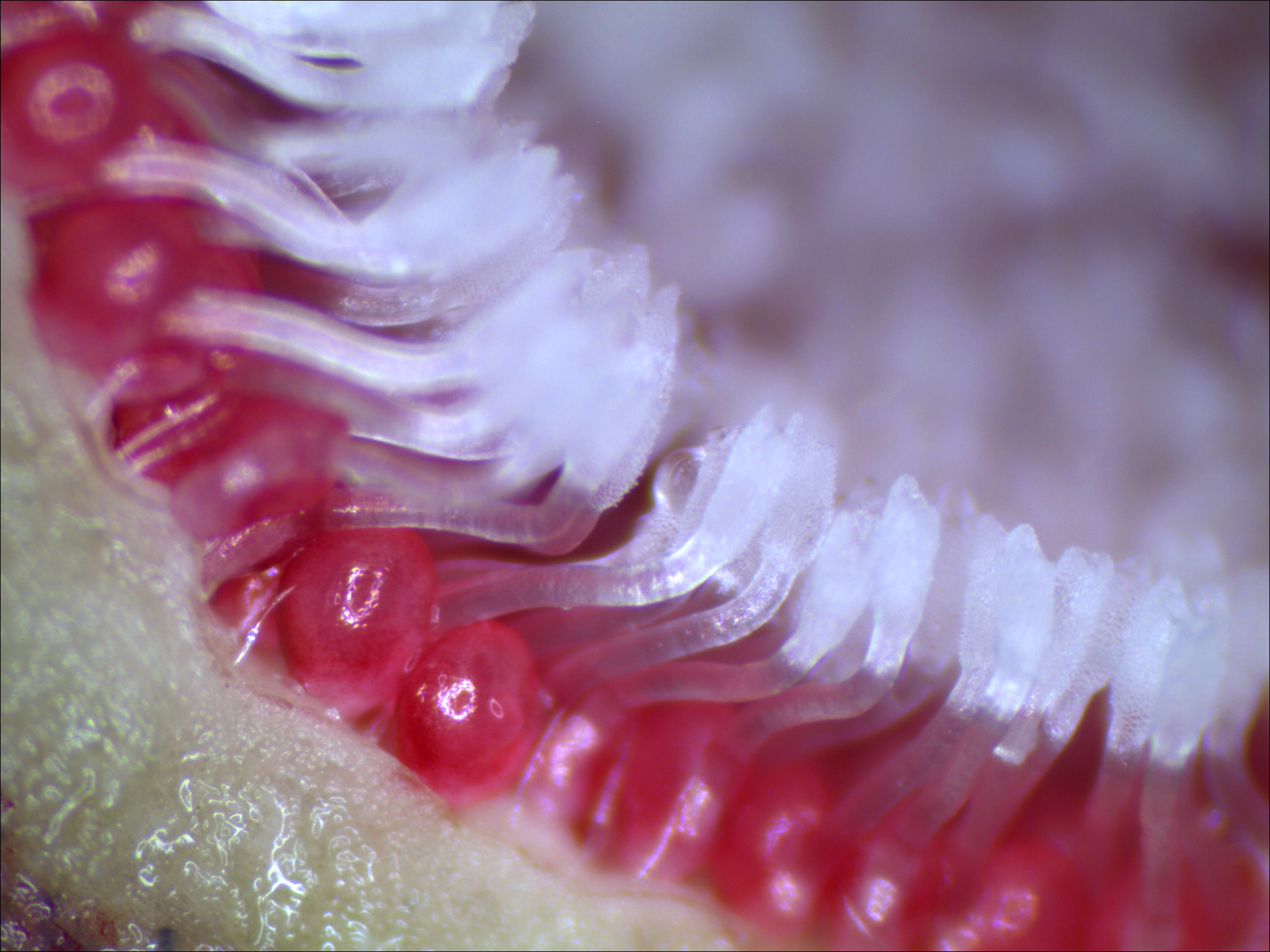


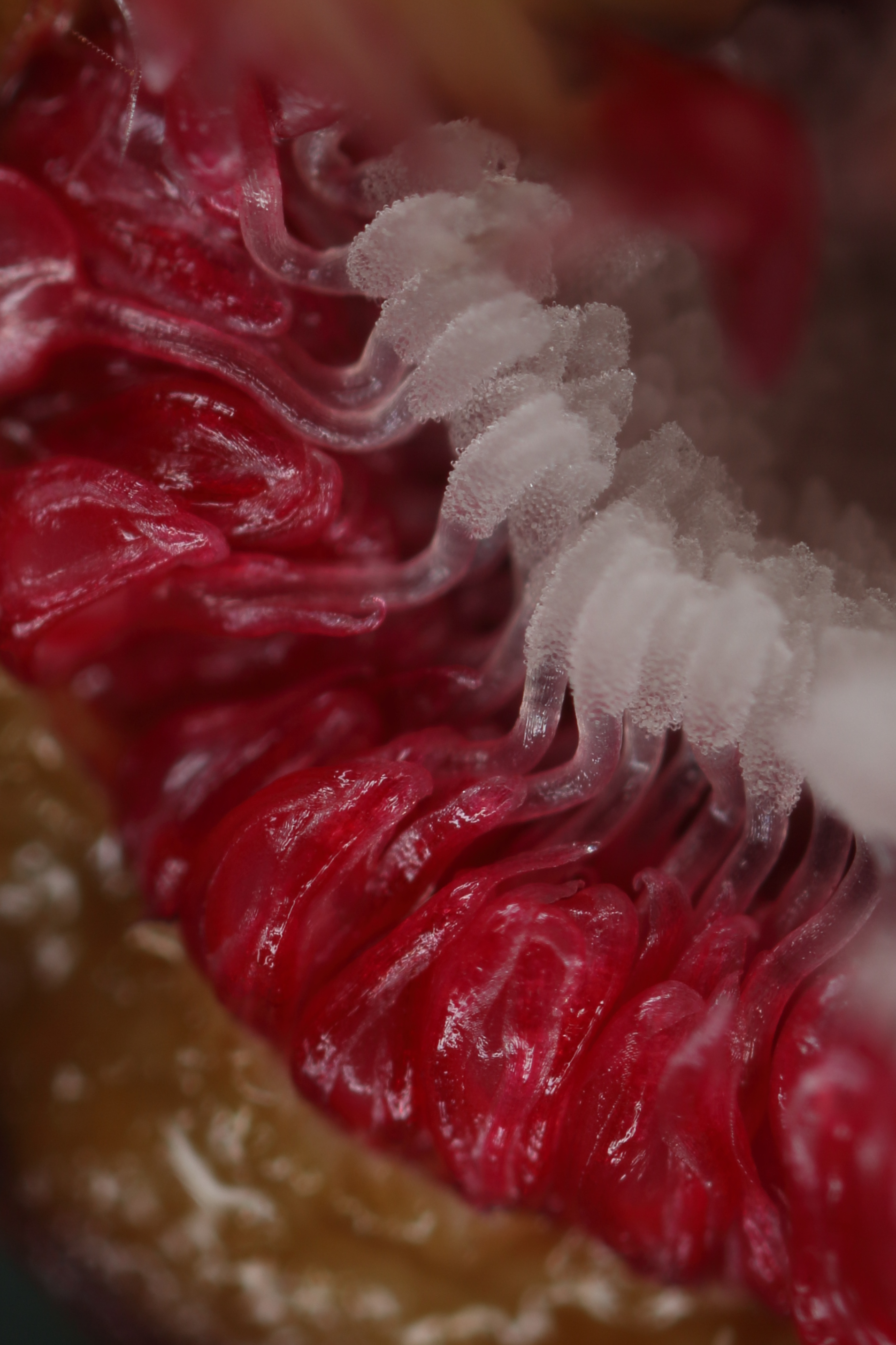


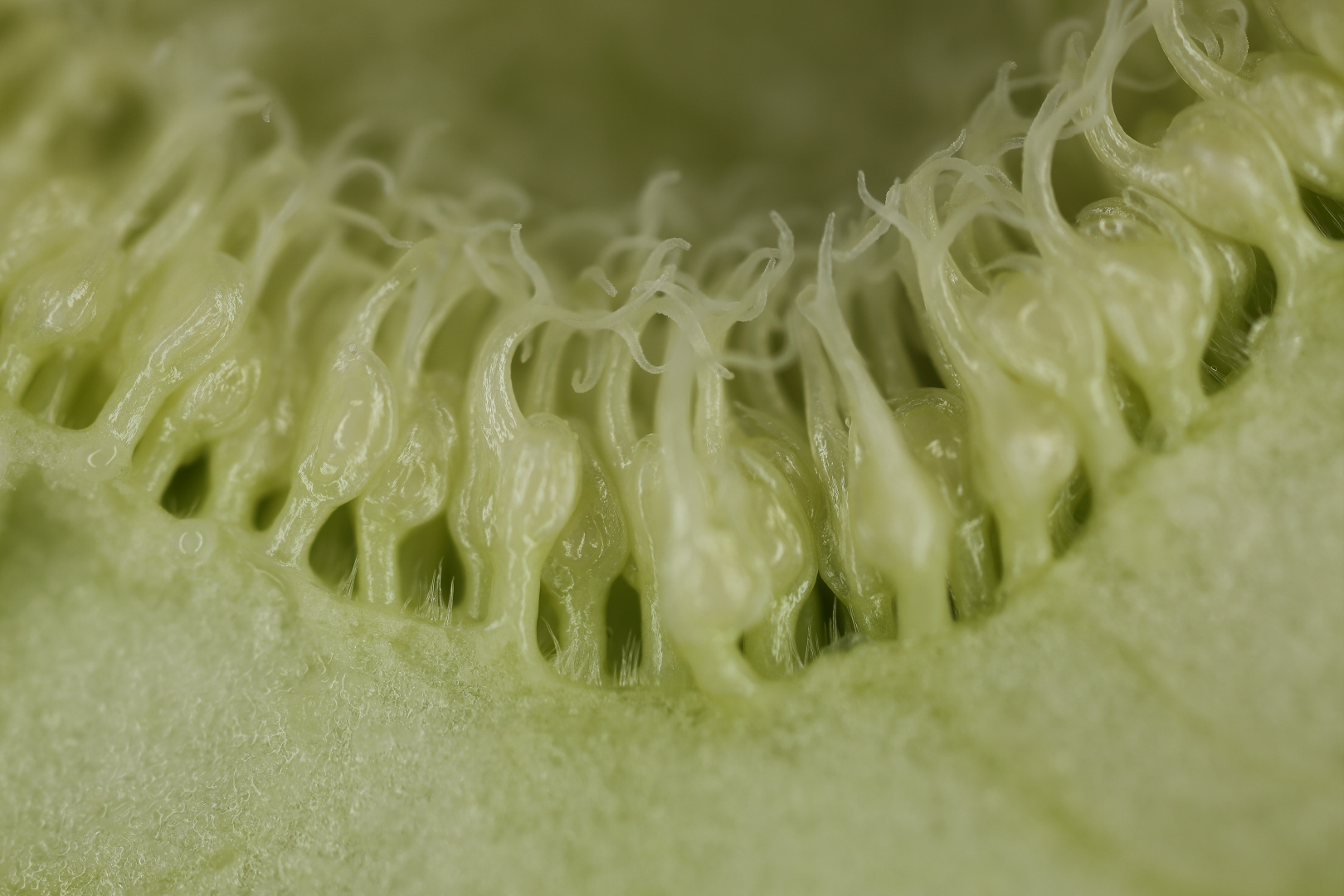


Fig. S2 The interiors of male figs collected in Mianyang (up) and Qujing (down), showing anther dehisced spontaneously before wasp emerging from the gall.


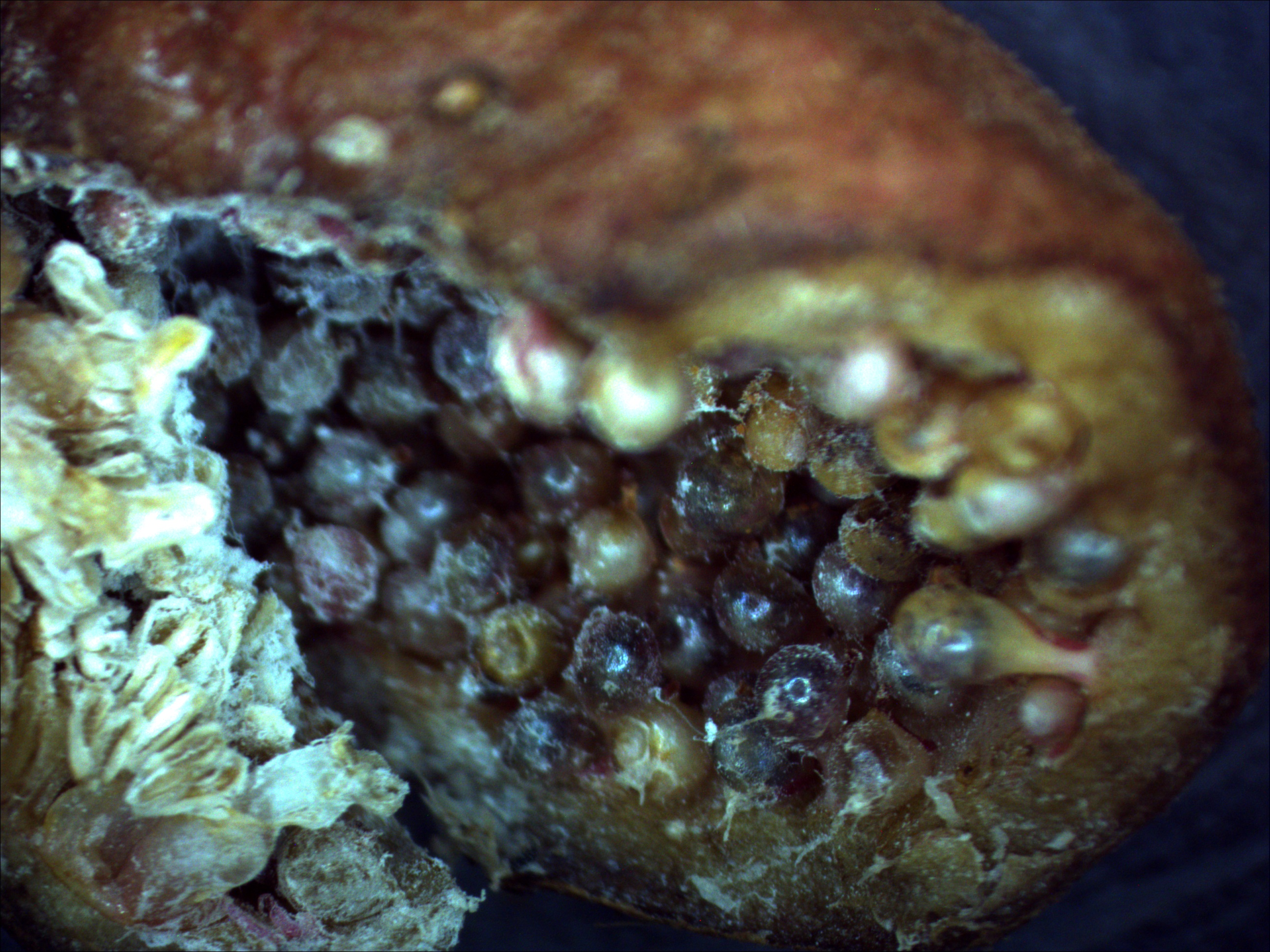


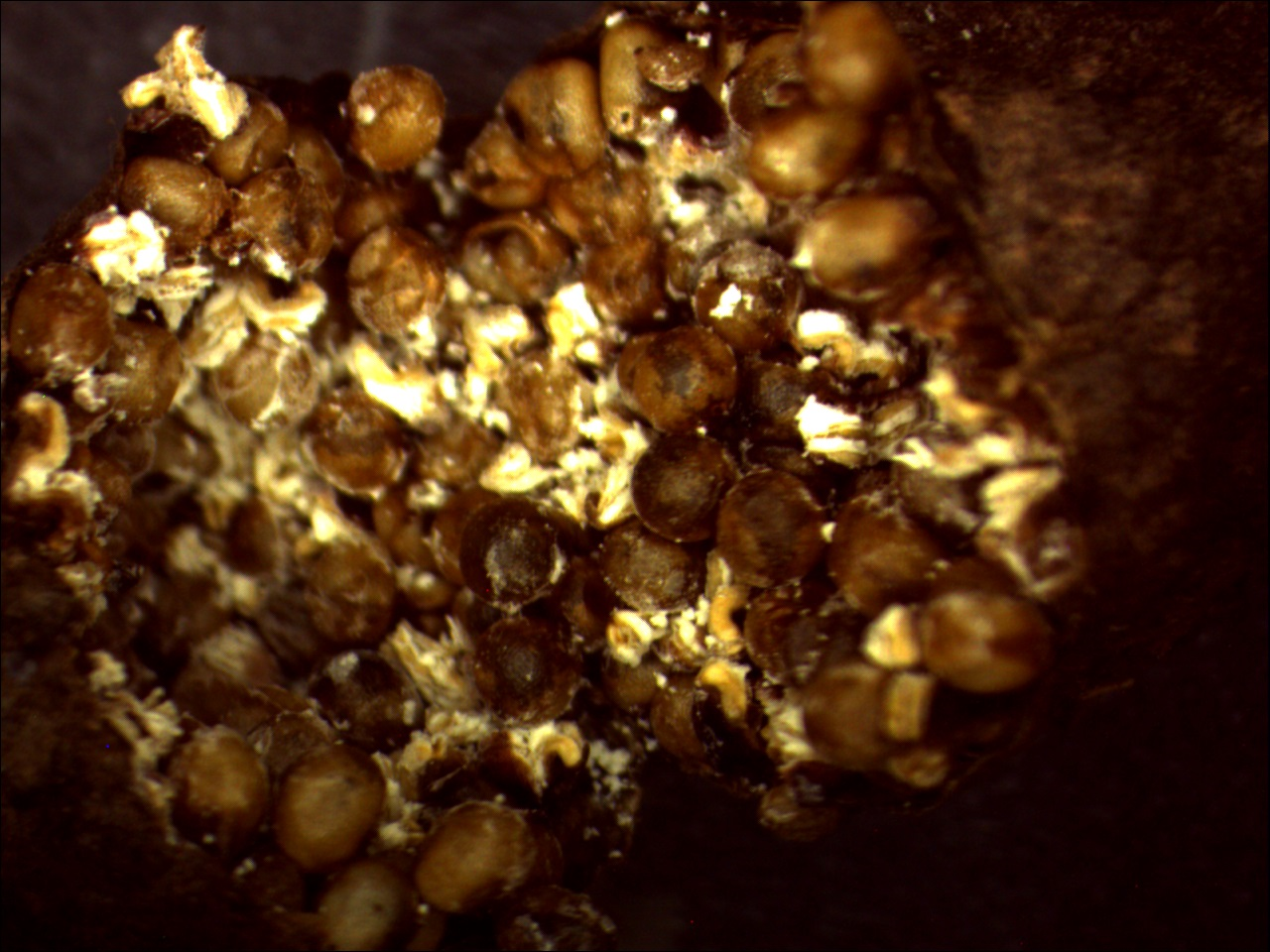

Supplement: Supplementary file 1 — Table S1. Comparisons of the numbers of sample squares (equivalent to individual plants) with different fig male flower distributions in the sampled populations of Ficus tikoua. Table S2. The flower numbers and anther‐to‐ovule (A/O) ratios in figs of Ficus tikoua from southwest China. Fig. S1. The interiors of female figs of Ficus tikoua (up), F. hainanensis (middle), and F. carica (down) at the receptive stage when pollinators enter. Fig. S2. The interiors of male figs collected in Mianyang (up) and Qujing (down), showing anther dehisced spontaneously before wasp emerging from the gall. [file ECE3-6-607-s001.docx]
